# A comparative study of ultrasound and cross-sectional imaging for detection of small renal masses: anatomic factors and radiologist's experience

**DOI:** 10.31744/einstein_journal/2020AO5576

**Published:** 2020-11-05

**Authors:** Fernando Ide Yamauchi, Omir Antunes Paiva, Thaís Caldara Mussi, Miguel José Francisco, Ronaldo Hueb Baroni

**Affiliations:** 1 Hospital Israelita Albert Einstein São PauloSP Brazil Hospital Israelita Albert Einstein, São Paulo, SP, Brazil.

**Keywords:** Ultrasonography, Diagnostic imaging, kidney neoplasms/diagnostic, imaging, Multidetector computed tomography

## Abstract

**Objective:**

To evaluate anatomic factors and radiologist's experience in the detection of solid renal masses on ultrasonography.

**Methods:**

We searched for solid renal masses diagnosed on cross-sectional imaging from 2007 to 2017 that also had previous ultrasonography from the past 6 months. The following features were evaluated: nodule size, laterality, location and growth pattern, patient body mass index and radiologist's experience in ultrasound. In surgically resected cases, pathologic reports were evaluated. Unpaired *t* test and *χ*^2^ test were used to evaluate differences among subgroups, using R-statistics. Statistical significance was set at p<0.05.

**Results:**

The initial search of renal nodules on cross-sectional imaging resulted in 428 lesions and 266 lesions were excluded. Final cohort included 162 lesions and, of those, 108 (67%) were correctly detected on ultrasonography (Group 1) and 54 (33%) were missed (Group 2). Comparison of Groups 1 and 2 were as follows, respectively: body mass index (27.7 *versus* 27.1; p=0.496), size (2.58cm *versus* 1.74cm; p=0.003), laterality (54% *versus* 59% right sided; p=0.832), location (27% *versus* 22% upper pole; p=0.869), growth pattern (25% *versus* 28% endophytic; p=0.131) and radiologist's experience (p=0.300). From surgically resected cases, histology available for Group 1 was clear cell (n=11), papillary (n=15), chromophobe (n=2) renal cell carcinoma, oncocytoma (n=1), and, for Group 2, clear cell (n=7), papillary (n=5) renal cell carcinoma, oncocytoma (n=2), angiomyolipoma, chromophobe renal cell carcinoma, and interstitial pyelonephritis (n=1, each).

**Conclusion:**

Size was the only significant parameter related to renal nodule detection on ultrasound.

## INTRODUCTION

Renal cell carcinoma (RCC) ranks ninth as most common type of cancer in males, and the incidence is rising particularly in developing countries, partly due to increase in established risk factors, and also due to widespread use of imaging modalities performed for other abdominal complaints.^(^^1^^-^^3^^)^ For those reasons, the majority of RCC are now incidentally diagnosed (over 50%), resulting in size and stage migration towards smaller RCC.

Several screening approaches have been debated, and most of them acknowledge the use of imaging modalities as part of those strategies.^(^^3^^-^^9^^)^ Data from other screening programs, such as for aortic aneurisms and colon cancer, using computed tomography (CT), have shown that renal lesions are a very common incidental finding (40% to 70%), but only a small fraction of those lesions are truly malignant renal neoplasms (0.21%).^(^^10^^,^^11^^)^ Therefore, although considered as gold standard not only for detection, but also for staging purpose, CT has several limitations for renal mass screening. There is significant burden on patients, with high incidence of indeterminate lesions diagnosed, that might need further investigation or follow-up, resulting in elevated financial resources and concern of using ionizing radiation.

Ultrasound (US) became a potential screening tool for renal masses, given low-cost, wide availability and lack of ionizing radiation. However, US is less sensitive and specific compared to CT for detecting renal masses, particularly in small lesions. The use of modern US equipment with tissue harmonics could further improve detection rate,^(^^12^^)^ but some factors, such as obesity, growth pattern, echogenicity and location could interfere in the detection on US.^(^^13^^,^^14^^)^

Several studies evaluated the role of US as screening tool for renal masses.^(^^5^^,^^7^^,^^8^^,^^15^^)^ and few studies compared US and CT accuracies,^(^^16^^,^^17^^)^ but focusing primarily on tumor size.

## OBJETIVE

To evaluate anatomic factors and radiologist's experience related to the detection of solid renal masses on ultrasonography.

## METHODS

We searched for solid renal masses diagnosed on CT or magnetic resonance imaging (MRI), from January 2007 to April 2017, using the terms “renal mass” or “renal nodule”, which had previous US performed within 6 months before CT. Ultrasound was performed by several radiologists from imaging department (more than ten) with different years and levels of experience, who were categorized into three groups: less than 5 years, 5 to 10 years and more than 10 years. A board-certified radiologist with 1-year experience in abdominal radiology retrospectively evaluated CT and MRI characteristics. The original radiology report was considered for nodule detection.

For CT or MRI, one of the authors evaluated the following aspects of the lesion: laterality, growth pattern (completely intrarenal/endophytic, partially exophytic – <50% – and exophytic – ≥50%), location (upper, middle, lower pole, upper and middle, middle and lower) and lesion size. Data on body mass index (BMI), age and gender were obtained from the patient records. In cases in which surgery was performed, pathology results were also recorded.

Welch *t* test was used for continuous variables and *χ*^2^ test for categorical variables to evaluate differences among subgroups, using R-statistics.

The study was approved by the Research Ethics Committee of *Hospital Israelita Albert Einstein* (HIAE), protocol # 3.722.121, CAAE: 16415619.0.0000.0071.

## RESULTS

The initial search of renal nodules on CT or MRI resulted in 428 lesions in the period. A total of 266 were excluded due to the following reasons: 256 with no previous US, and 10 with missing information on BMI. The final cohort included 162 lesions: 67% were correctly detected on the previous ultrasound (108/162), categorized as Group 1, and 33% were missed (54/162), categorized as Group 2. Comparative analysis of Groups 1 and 2 is summarized on table 1.

**Table 1:** Comparative analysis of lesions detected on ultrasonography (Group 1) and lesions missed on ultrasonography (Group 2) in absolute (and relative - %) values

	Group 1	Group 2	p value
	(n=108)	(n=54)	
Laterality, n (%)	Right: 58 (54)	Right: 32 (59)	0.832
	Left: 50 (46)	Left: 22 (41)	
Location, n (%)	Upper: 29 (27)	Upper: 12 (22)	0.869
	Middle: 39 (36)	Middle: 21 (39)	
	Lower: 27 (25)	Lower: 16 (30)	
	Upper and middle: 7 (6)	Upper and middle: 4 (7)	
	Middle and lower: 6 (6)	Middle and lower: 1 (2)	
Growth pattern, n (%)	Endophytic: 27 (25)	Endophytic: 15 (28)	0.131
	Partially exophytic: 57 (53)	Partially exophytic: 33 (61)	
	Exophytic: 24 (22)	Exophytic: 6 (11)	
Size, cm	2.58 (range: 0.3-9.0)	1.74 (range: 0.4–4.0)	0.003
BMI	27.7	27.1	0.496

BMI: body mass index.

Detection rate among the groups of radiologists with different experience (less than 5 years, 5 to 10 years ande more than 10 years) showed no statistically significant difference (p=0.300).

A total of 44 lesions were surgically resected (27%, 44/162). Histology of Group 1 was: clear cell RCC (n=11), papillary RCC (n=15), chromophobe RCC (n=2) and oncocytoma (n=1). Histology of Group 2 was: clear cell RCC (n=7), angiomyolipoma (n=1), chromophobe RCC (n=1), papillary RCC (n=5), oncocytoma (n=2) and interstitial pyelonephritis (n=1).

There were also 34 lesions that showed typical features of angiomyolipoma on CT or MRI, consequently considered benign and not submitted to any invasive treatment. The remaining lesions (84/162) had no final diagnosis established, since patients had not been submitted to any invasive treatment at our organization.

## DISCUSSION

The incidence of incidentally detected renal mass is rising, particularly due to widespread use of imaging modalities.^(^^1^^-^^3^^)^ While a migration toward smaller and earlier stage RCC is desirable, several screening strategies have been debated. Although CT and MRI have higher accuracies, investigating and following all focal renal lesions results in elevated costs and patient anxiety.^(^^10^^,^^18^^)^ In this scenario, US seems to fit properly, due to wide availability, relatively lower cost and no use of ionizing radiation.^(^^5^^)^

We evaluated which clinical and anatomical factors could influence the detection of solid renal masses on US. Several hypotheses have been suggested based on the common sense of radiology ultrasound daily practice: the left kidney is slightly higher than the right and the spleen offers smaller and worse acoustic window on US; smaller and more endophytic nodules are more difficult to detect; BMI could pose additional technical challenges and level of experience performing US could interfere in the detection rate.

However, only tumor size was associated to detection on US in our cohort. Several articles have already demonstrated that US lacks sensitivity in the evaluation of small renal masses.^(^^16^^,^^17^^,^^19^^)^ Interestingly, other tumor features such as laterality, location, growth pattern, BMI and radiology experience did not influence the detection of solid renal nodules on US in our study. We raised the hypothesis that modern US equipment might overcome difficulties in evaluation of intrarrenal lesions, as well as limitations of US performed on obese patients.

There are some limitations in this study. First, potential false negative US scans were missed due to selection bias (since patients included needed US and cross-sectional images). A prospective study could further confirm our hypothesis. Second, US was performed in our organization with highly trained radiologists, using modern US equipment, which may not reflect the daily practice in other facilities. Perhaps different results could be obtained in places where a technologist performs US, regarding experience, patient's BMI and tumor features. Third, clinical indication for US was not evaluated, and could potentially interfere with nodule detection (such as the evaluation of hematuria), since radiologists were aware of clinical information during the US examination. Forth, half of the lesions did not have a final diagnosis.

## CONCLUSION

Size is the only significant parameter related to renal nodule detection on ultrasound. Other features related to patient's body mass index, to the lesion (laterality, location and growth pattern) and to radiologist's experience were not associated to lesion detection.
