# Supplementary material for: A comparative study of ultrasound and cross-sectional imaging for detection of small renal masses: anatomic factors and radiologist's experience
Source: Einstein (Sao Paulo). 2020 Nov 5;18:eAO5576. doi: 10.31744/einstein_journal/2020AO5576 (PMC7647384; doi:10.31744/einstein_journal/2020AO5576)
Supplement: Translation

# Translation: Estudo comparativo de ultrassonografia e métodos seccionais para detecção de pequenas lesões renais: fatores anatômicos e experiência do radiologista

**Authors:** Fernando Ide Yamauchi, Omir Antunes Paiva, Thaís Caldara Mussi, Miguel José Francisco, Ronaldo Hueb Baroni

**Affiliations:** 2 São PauloSP Brasil Hospital Israelita Albert Einstein, São Paulo, SP, Brasil.

**Keywords:** Ultrassonografia, Diagnóstico por imagem, Neoplasias renais/diagnóstico por imagem, Tomografia computadorizada multidetectores

## Abstract

**Objetivo:**

Avaliar os fatores anatômicos e a experiência do radiologista na detecção de massas renais sólidas na ultrassonografia.

**Métodos:**

Buscamos massas renais sólidas diagnosticadas em imagens seccionais, de 2007 a 2017, que também tivessem ultrassonografia prévia nos últimos 6 meses. As seguintes características foram avaliadas: tamanho do nódulo, lateralidade, localização e padrão de crescimento, índice de massa corporal do paciente e experiência do radiologista em ultrassonografia. Nos casos com ressecção cirúrgica, os laudos de patologia foram analisados. O teste *t* não pareado e o teste *χ*^2^ foram utilizados para avaliar as diferenças entre os subgrupos, usando *R-statistics*. A significância estatística foi estabelecida em p<0,05.

**Resultados:**

A pesquisa inicial de nódulos renais achados em imagens seccionais resultou em 428 lesões, com 266 exclusões. A coorte final incluiu 162 lesões e, destas, 108 (67%) foram detectadas corretamente na ultrassonografia (Grupo 1), e 54 (33%) não foram identificadas (Grupo 2). A comparação dos Grupos 1 e 2 mostrou índice de massa corporal (27,7 *versus* 27,1; p=0,496), tamanho (2,58cm *versus* 1,74cm; p=0,003), lateralidade (54% *versus* 59% no lado direito; p=0,832), localização (27% *versus* 22% no polo superior; p=0,869), padrão de crescimento (25% *versus* 28% endofítico; p=0,131) e experiência do radiologista (p=0,300). A histologia disponível para o Grupo 1 foi carcinoma renal de células claras (n=11), papilar (n=15), cromófobo (n=2), oncocitoma (n=1), e, para o Grupo 2, carcinoma renal de células claras (n=7), papilar (n=5), oncocitoma (n=2), angiomiolipoma, cromófobo e pielonefrite intersticial (n=1, cada).

**Conclusão:**

O tamanho foi o único parâmetro significativo relacionado à detecção de nódulos renais no ultrassom.

## INTRODUÇÃO

O carcinoma de células renais (CCR) é o nono câncer mais comum em homens, e a incidência se eleva principalmente nos países em desenvolvimento, em parte devido ao aumento dos fatores de risco conhecidos e ao amplo uso de métodos de imagem realizados para outras queixas abdominais.^(^^1^^-^^3^^)^ Por essas razões, a maioria dos CCR é hoje diagnosticada de forma incidental (acima de 50%), resultando em migração para CCR menores em tamanho e estadiamento.

Várias abordagens de rastreamento têm sido debatidas, e a maioria reconhece o uso de exames de imagem como parte dessas estratégias.^(^^3^^-^^9^^)^ Dados de outros programas de rastreamento, por exemplo, para aneurismas de aorta e câncer de cólon usando tomografia computadorizada (TC) mostraram que lesões renais são achados incidentais muito comuns (40% a 70%), mas apenas uma pequena fração dessas lesões é verdadeiramente neoplasia renal maligna (0,21%).^(^^10^^,^^11^^)^ Assim, embora considerada padrão-ouro não apenas para detecção, mas também para fins de estadiamento, a TC tem várias limitações para o rastreamento de massa renal. Existe alta incidência de diagnósticos de lesões indeterminadas, que podem precisar de novas investigações ou de seguimento, resultando em recursos financeiros elevados e em preocupação com o uso de radiação ionizante.

A ultrassonografia (US) tornou-se ferramenta potencial de rastreamento para massas renais em função do baixo custo, da ampla disponibilidade e do não uso de radiação ionizante. No entanto, a US é menos sensível e específico para a detecção de massas renais, se comparado à TC, particularmente em se tratando de pequenas lesões. O uso de equipamentos modernos de US com harmônica tecidual poderia melhorar ainda mais a taxa de detecção,^(^^12^^)^ mas alguns fatores, como obesidade, padrão de crescimento, ecogenicidade e localização podem interferir na detecção pela US.^(^^13^^,^^14^^)^

Vários estudos avaliaram o papel da US como ferramenta de rastreamento para massas renais,^(^^5^^,^^7^^,^^8^^,^^15^^)^ e alguns poucos compararam a precisão de US e TC,^(^^16^^,^^17^^)^ mas focaram primariamente no tamanho do tumor.

## OBJETIVO

Avaliar os fatores anatômicos e a experiência do radiologista relacionados à detecção de lesões renais sólidas na ultrassonografia.

## MÉTODOS

Fizemos a busca por massas renais sólidas diagnosticadas em TC ou ressonância magnética (RM), de janeiro de 2007 a abril de 2017, usando os termos “massa renal” ou *“*nódulo renal”, que tivessem US realizado nos 6 meses anteriores à TC ou RM. A US foi realizada por vários radiologistas do departamento de imagem (mais de dez), com anos e níveis de experiência diferentes, que foram classificados em três grupos: menos de 5 anos, 5 a 10 anos e mais de 10 anos. Um radiologista com título de especialista com 1 ano de experiência em radiologia abdominal avaliou retrospectivamente as características analisadas em TC e RM. O laudo radiológico original foi considerado para detecção de nódulos.

Para TC ou RM, um dos autores avaliou os seguintes aspectos da lesão: lateralidade, padrão de crescimento (completamente intrarrenal/endofítico, parcialmente exofítico – <50% – e exofítico – ≥50%), localização (polo superior, médio, inferior, superior e médio, médio e inferior) e tamanho da lesão. Dados sobre índice de massa corpórea (IMC), idade e gênero foram obtidos nos prontuários dos pacientes. Nos casos em que foi realizada cirurgia, os resultados da patologia também foram registrados.

O teste t de Welch foi utilizado para variáveis contínuas e o teste *χ*^2^ para variáveis categóricas para avaliar diferenças entre os subgrupos, usando a estatística R.

O estudo foi aprovado pelo Comitê de Ética em Pesquisa do Hospital Israelita Albert Einstein (HIAE), sob parecer número 3.722.121, CAAE: 16415619.0.0000.0071.

## RESULTADOS

A busca inicial de nódulos renais em TC ou RM resultou em 428 lesões no período. Foram excluídos 266 pelos seguintes motivos: 256 sem US anterior e dez sem informações sobre o IMC. A coorte final incluiu 162 lesões: 67% foram detectadas corretamente na US anterior (108/162), categorizadas como Grupo 1, e 33% foram perdidas (54/162), categorizadas como Grupo 2. A análise comparativa dos Grupos 1 e 2 está resumida na tabela 1.

**Tabela 1:** Análise comparativa das lesões detectadas na ultrassonografia (Grupo 1) e lesões não encontradas na ultrassonografia (Grupo 2) em valores absolutos (e relativos - %)

	Grupo 1	Grupo 2	Valor de p
	(n=108)	(n=54)	
Lateralidade, n (%)	Direito: 58 (54)	Direito: 32 (59)	0,832
	Esquerdo: 50 (46)	Esquerdo: 22 (41)	
Localização, n (%)	Superior: 29 (27)	Superior: 12 (22)	0,869
	Médio: 39 (36)	Médio: 21 (39)	
	Inferior: 27 (25)	Inferior: 16 (30)	
	Superior e médio: 7 (6)	Superior e médio: 4 (7)	
	Médio e inferior: 6 (6)	Médio e inferior: 1 (2)	
Padrão de crescimento, n (%)	Endofítico: 27 (25)	Endofítico: 15 (28)	0,131
	Parcialmente exofítico: 57 (53)	Parcialmente exofítico: 33 (61)	
	Exofítico: 24 (22)	Exofítico: 6 (11)	
Tamanho, cm	2,58 (variação: 0,3-9,0)	1,74 (variação: 0,4-4,0)	0,003
IMC	27,7	27,1	0,496

IMC: índice de massa corpórea.

A taxa de detecção entre os grupos de radiologistas com diferentes experiências (menos de 5 anos, 5 a 10 anos e mais de 10 anos) não apresentou diferença com significância estatística (p=0,300).

Foram ressecadas cirurgicamente 44 (27%, 44/162) lesões. A histologia do Grupo 1 foi CCR de células claras (n=11), CCR papilar (n=15), CCR cromófobo (n=2) e oncocitoma (n=1). No Grupo 2, a histologia foi a seguinte: CCR de células claras (n=7), angiomiolipoma (n=1), CCR cromófobo (n=1), CCR papilar (n=5), oncocitoma (n=2) e pielonefrite intersticial (n=1).

Havia também 34 lesões que apresentavam características típicas do angiomiolipoma na TC ou RM, consequentemente consideradas benignas e não submetidas a nenhum tratamento invasivo. As demais lesões (84/162) não tiveram diagnóstico final estabelecido, pois os pacientes não foram submetidos a nenhum tratamento invasivo em nossa instituição.

## DISCUSSÃO

A incidência de massa renal detectada incidentalmente tem aumentado, em especial devido ao uso generalizado de exames de imagem.^(^^1^^-^^3^^)^ Várias estratégias de rastreamento têm sido debatidas, focando em estadiamentos de CCR mais precoces. Embora a TC e a RM tenham maior precisão, investigar e acompanhar todas as lesões renais focais resultam em custos elevados e em ansiedade para o paciente.^(^^10^^,^^18^^)^ Nesse cenário, a US parece ser adequado devido a sua ampla disponibilidade e seu custo relativamente menor e por não utilizar radiação ionizante.^(^^5^^)^

Avaliamos quais fatores clínicos e anatômicos podem influenciar na detecção de massas renais sólidas na US. Várias hipóteses foram sugeridas com base no bom senso da prática diária de US pela radiologia: o rim esquerdo é um pouco mais alto que o direito, e o baço oferece uma janela acústica menor e pior na US; nódulos menores e mais endofíticos são mais difíceis de detectar; o IMC pode representar desafios técnicos adicionais e o nível de experiência na realização de US pode interferir na taxa de detecção.

Entretanto, em nossa coorte, apenas o tamanho do tumor foi associado à detecção na US. Vários artigos já demonstraram que a US tem pouca sensibilidade para avaliação de massas renais pequenas.^(^^16^^,^^17^^,^^19^^)^ É interessante que outras características do tumor, como lateralidade, localização, padrão de crescimento, IMC e experiência em radiologia, não influenciaram na detecção de nódulos renas sólidos pela US em nosso estudo. Nossa hipótese é a de que equipamentos modernos de US podem superar dificuldades na avaliação de lesões intrarrenais e, inclusive, limitações da US realizada em pacientes obesos.

Existem algumas limitações neste estudo. Primeiro, os potenciais exames de US falsos-negativos foram perdidos devido ao viés de seleção (uma vez que os pacientes incluídos deveriam ter US e imagens seccionais). Um estudo prospectivo poderia confirmar nossa hipótese. Segundo, as ultrassonografias foram realizadas em nossa instituição por radiologistas altamente treinados e em modernos equipamentos de US, que podem não refletir a prática diária em outras instituições. Talvez nos locais onde um tecnólogo realiza o exame, resultados diferentes possam ser obtidos em relação à experiência, ao IMC do paciente e às características do tumor. Terceiro, a indicação clínica para US não foi avaliada e poderia potencialmente interferir na detecção de nódulos (como avaliação de hematúria), uma vez que os radiologistas estavam cientes das informações clínicas durante o exame de US. Quarto, metade das lesões não teve diagnóstico final confirmado por patologia.

## CONCLUSÃO

O tamanho foi o único parâmetro significativo relacionado à detecção de nódulos renais pela ultrassonografia. Outras características relacionadas ao índice de massa corporal, à lesão (lateralidade, localização e padrão de crescimento) e à experiência do radiologista não foram associadas à detecção de lesão.
